# Case Report: Fabry disease overlapping with systemic lupus erythematosus in a pediatric patient

**DOI:** 10.3389/fimmu.2025.1620776

**Published:** 2025-09-01

**Authors:** Yaqing Liu, Juanjuan Luo, Nengjing Wu, Kaiyuan Luo, Jialing Liu, Liangzhong Sun

**Affiliations:** ^1^ Department of Pediatrics, First Affiliated Hospital, Gannan Medical University, Ganzhou, JiangXi, China; ^2^ Department of Pediatrics, Aviation General Hospital, Beijing, China; ^3^ Department of Pediatrics, Nanfang Hospital, Southern Medical University, Guangzhou, China

**Keywords:** Fabry disease, systemic lupus erythematosus, enzyme replacement therapy, hydroxychloroquine, belimumab, GLA

## Abstract

Fabry disease (FD) is an X-linked lysosomal storage disease caused by a deficiency of the enzyme alpha-galactosidase (α-Gal). Systemic lupus erythematosus (SLE) is a chronic autoimmune disease with multisystem involvement and predominantly affects women of childbearing age. FD and SLE affect similar organs and may show overlapping features. However, the coexistence of FD with SLE is an infrequent incidence. A 12-year-old Chinese boy was diagnosed to have SLE based on the symptoms of fever, glomerular hematuria, nephrotic-range proteinuria, hypocomplementaemia, and positivity for antinuclear antibodies and anti-double-stranded deoxyribonucleic acid antibodies. Light microscopy of kidney biopsy samples revealed characteristic features of SLE (Classification IV+V). Additionally, electron microscopy of the biopsy samples demonstrated osmiophilic myelin-like bodies in the cytoplasm of glomerular podocytes.The leukocytic α-GLA activity was abnormally low. Genetic analysis showed that the patient was hemizygous for the c.G735C mutation in exon 5 of the GLA gene, which was inherited from his mother and maternal grandmother who were heterozygous and asymptomatic. Hydroxychloroquine (HCQ) administration was discontinued based on renal pathological examination results. The patient was commenced on methylprednisolone pulses and intravenous cyclophosphamide administration, followed by maintenance therapy. He was also treated with angiotensin-converting enzyme inhibitors and an angiotensin receptor blocker. This treatment regimen led to only partial improvement in the patient’s condition. Enzyme replacement therapy (ERT) with agalsidase-α (0.2 mg/kg intravenous administration every 2 weeks) was initiated 2 months after diagnosis. The complement levels remained persistently low. Moreover, treatment with belimumab failed to improve the levels of serological markers. Following the comprehensive treatment regimen, the proteinuria levels remained stable at below 500 mg/24 h. To the best of our knowledge, we report here the case of the youngest patient with a novel FD-related mutation coexistent with SLE. Renal biopsy plays a critical role as an indicator of FD coexisting with nephropathy. Furthermore, genetic testing could serve as a crucial assessment, particularly for male patients with SLE. This case report also addressed the controversial issue of HCQ use in patients with coexistent FD and SLE, examined the effect of ERT on proteinuria, and assessed the role of complement activation in disease progression and treatment.

## 1 Introduction

Systemic lupus erythematosus (SLE) is a chronic autoimmune inflammatory disorder characterized by multisystem and multiorgan involvement, and it exhibits remarkable heterogeneity (1). Childhood-onset SLE (cSLE), defined as SLE diagnosed before the age of 18 years, constitutes approximately 15%–20% of all SLE cases (2). cSLE typically begins between ages 12 and 14, predominantly in females, and presents more severe symptoms than adult SLE, particularly affecting the kidneys, blood, and nervous system (3). Fabry disease (FD) is a rare X-linked genetic disorder caused by mutations affecting α-galactosidase A, leading to a deficiency in enzyme activity and impacting multiple organs (4). Its occurrence ranges from 1 in 1250 to 1 in 8882 newborns (5–7) with a study from Mainland China showing a 0.12% prevalence (8). Most males with FD display classic symptoms like limb pain, skin lesions, abnormal sweating, eye opacities, and proteinuria in childhood or adolescence, often leading to end-stage renal disease by age 30. The coexistence of FD and SLE is very rare, with only a few adult cases reported.We conducted a retrospective analysis of the clinical data of a male patient who was diagnosed with FD and SLE at the age of 12. Through a comprehensive literature review, we synthesized the clinical characteristics and genetic mutation profiles of patients exhibiting both conditions. In the final discussion, we explored the pathogenesis underlying the coexistence of these diseases and deliberated several uncertainties associated with the treatment.

## 2 Case report

A male patient was admitted on November 7, 2021, with a complaint of recurrent fever for 10 days. He also presented with hematuria, proteinuria, and a tendency to develop chilblains in winter. Born to non-consanguineous parents via vaginal delivery at 39 weeks, the patient weighed 3 kg at birth. The mother had recurrent oral ulcers, while the rest of the family, except for a maternal uncle who died at age 20 from an unspecified brain disease, is healthy. A comprehensive review of the patient’s family medical history revealed no definitively confirmed cases of autoimmune disorders among first- or second-degree relatives.

## 3 Physical examination of the patient at admission

Upon admission, the patient had a temperature of 38.6°C, pulse of 132 bpm, respiratory rate of 22 breaths/min, and blood pressure of 108/65 mmHg. The patient, who was 143 cm tall and weighed 36.5 kg (both in the 10th–25th percentile), was conscious with no rash or petechiae. Cardiopulmonary, abdominal, and neurological exams were normal, but mild pitting edema was present in the lower legs. The spine was normal, with no joint issues.

## 4 Laboratory and imaging examinations

Lab tests showed mild anemia (hemoglobin 101 g/L) but normal white blood cell and platelet counts. Blood biochemistry revealed urea nitrogen at 5.4 mmol/L, creatinine at 106 μmol/L, eGFR at 65 mL/min/1.73m², albumin at 22.3 g/L, and serum potassium at 3.2 mmol/L. Liver and myocardial enzymes, as well as blood lipids, were normal. The following values were recorded for infection and inflammation parameters: procalcitonin at 0.29 ng/mL, interleukin-6 at 5.35 pg/mL, C-reactive protein below 0.8 mg/L, erythrocyte sedimentation rate at 50 mm/h, and Epstein-Barr virus DNA at 5.47 × 10³ copies/mL(reference range:<500copies/mL).Tests for T-spot.TB, Mycoplasma pneumoniae, and cytomegalovirus were negative. Immunological tests showed IgG at 29.48 g/L, IgA at 4.11 g/L, IgM at 1.61 g/L, C3 at 6.9 mg/dL, C4 at 1.3 mg/dL, and antinuclear antibodies (ANA) at 1:1000 (+)(EUROIMMUN-Specific System). Positive results were found for anti-ds-DNA, anti-SSA, anti-SSB, and anti-Ro-52 antibodies, while lupus anticoagulant and anticardiolipin antibodies were negative. Soluble C5b-9 levels were elevated at 1179 ng/mL(reference range:75-219ng/mL), indicating complement system overactivation. Lymphocyte subset analysis showed total T lymphocytes at 84.74%(absolute count:1204 cells/μL), helper/inducer T cells at 28.96% (absolute count:511cells/μL), suppressor/cytotoxic T cells at 51.56% (absolute count:909cells/μL), double-positive T cells at 0.34% (absolute count:6cells/μL), double-negative T cells at 4.1% (absolute count:58cells/μL), a helper/suppressor T cell ratio of 0.56, and B cells at 11.99% (absolute count:130cells/μL) and NK cells at 3.04%(absolute count:33cells/μL). 25-hydroxyvitamin D [25(OH)D]was 16.51 ng/mL(reference range:≧30ng/mL), and cancer antigen 125 was 57.7 U/mL(reference range: ≤24 U/mL). Urine sediment microscopy revealed 12–23 red blood cells (RBCs) per high-power field (HPF), 0–3 white blood cells (WBCs) per HPF, no renal tubular epithelial cells, no calcium oxalate, uric acid, cysteine or ammonium phosphate crystals, 2–4 hyaline casts per HPF, and no other casts of pathological significance. Measurement of 24-hour urinary protein revealed 7.94 g/day. Bone marrow examination showed active cell proliferation, reduced erythroblasts, and no tumor cells. Imaging revealed small pleural, pericardial, peritoneal, and pelvic effusions. High-resolution computed tomography(HRCT)of the chest showed interstitial pulmonary edema and bilateral pneumonia. Pulmonary tests indicated moderate restrictive dysfunction and severe diffusion impairment. One-year follow-up HRCT revealed residual linear opacities in the left lower lobe, indicating post-inflammatory changes. Pulmonary function tests showed progressive improvement: restrictive dysfunction decreased from moderate to mild at 1 month, with diffusion impairment improving from severe to moderate.The diffusing capacity (DLCO) showed mild impairment at 66.7% of predicted during the 6months follow-up.Cranial magnetic resonance imaging combined with magnetic resonance angiography showed no abnormalities. Ophthalmological and hearing assessments showed normal results.

## 5 Renal histopathology

**Figure 1:**
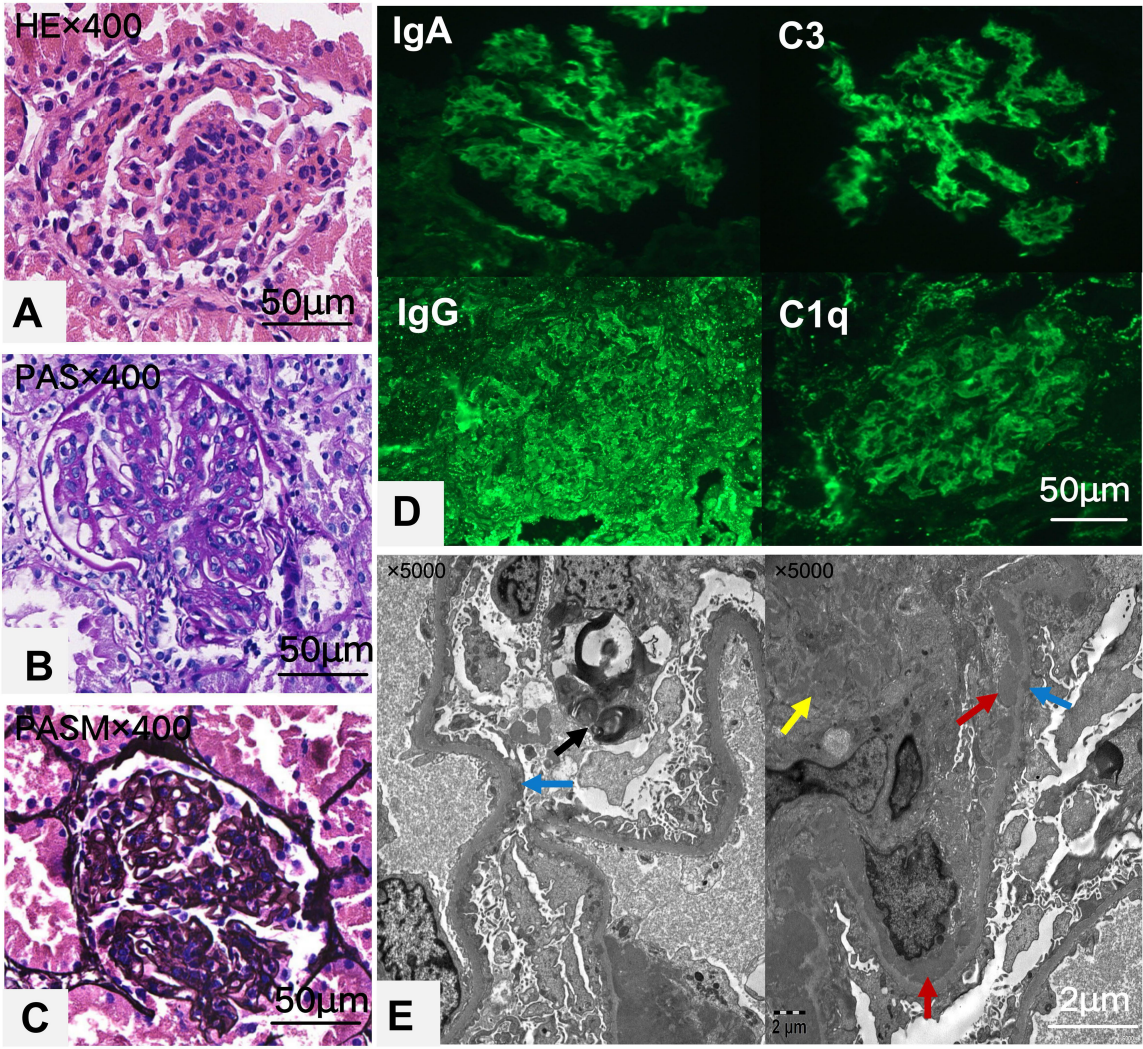
Histological and immunofluorescence images of renal tissue are shown in a series of panels. Panel **(A)** shows an H&E stained section, highlighting glomerular inflammation. Panel **(B)** displays a PAS stain, enhancing the extracellular matrix details. Panel **(C)** shows a PASM stain, showing basement membrane features. Panel **(D)** provides immunofluorescence images for IgA, IgG, C3, and C1q, indicating immunoglobulin and complement deposits. Panel **(E)** features an electron micrograph with arrows indicating electron-dense deposits in the subendothelial(red),mesangial(yellow) and subepithelial areas(blue), with myeloid bodies(black) in occasional podocytes. Each image includes a scale bar for size reference.

Light microscopy reveals 13 glomeruli, with no global or segmental glomerulosclerosis observed. Diffuse moderate to severe mesangial cell and matrix proliferation and endothelial cell proliferation were noted in the glomeruli (**Figures 1A-C**). Neutrophil infiltration and karyorrhexis were observed within the glomeruli, with partial capillary compression. The subepithelial, subendothelial, and mesangial areas showed deposition of eosinophilic proteins, with the formation of a few hyaline structures. Segmental double-track changes were visible, and individual capillary loops exhibited microthrombus formation. Parietal epithelial cells did not show proliferation or crescent formation. Immunofluorescence assays revealed widely distributed spherical deposits of immunoglobulin G (IgG) (3+),immunoglobulin A (IgA) (3+), immunoglobulin M(IgM) (1+), C3 (3+), and C1q (3+) localized in the mesangial area and capillary walls(**Figure 1D**). These findings corroborated the diagnosis of lupus nephritis (International Society of Nephrology/Renal Pathology Society Classification IV-A+V) with an activity index of 6 and a chronicity index of 0. These findings were further confirmed by electron microscopy, which showed mesangial cell and matrix proliferation; electron-dense deposits localized in the subepithelial, subendothelial, basement membrane, and mesangial regions; cytoplasmic swelling and vacuolar degeneration in capillary endothelial cells and podocytes; and presence of a few myeloid bodies in podocytes. No organized deposits (e.g., fibrillary/fingerprint patterns) or endothelial tubuloreticular inclusions were identified by electron microscopy (**Figure 1E**).

## 6 Enzymatic activity and genetic testing

**Figure 2:**
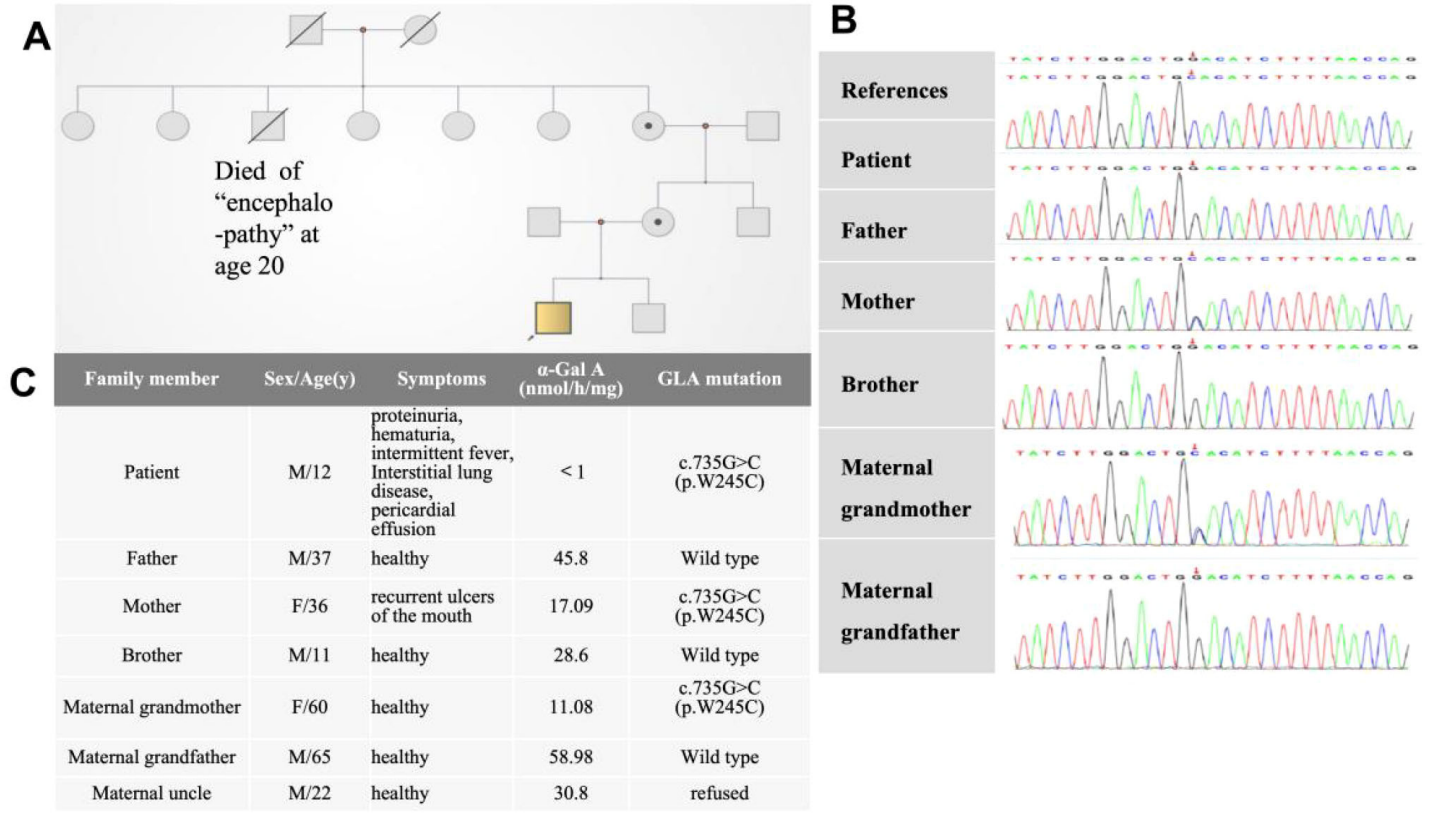
**(A)** Pedigree chart and **(B)** sequencing results of the GLA mutations. **(C)** Clinical findings of family members.

The α-Gal A activity levels in the patient, his mother, and maternal grandmother were below the reference range(>24.7nmol/h/mg, Colorimetric assay) with the patient showing the lowest enzymatic activity (<1.0 nmol/h/mg). In contrast, the α-Gal A activity levels in the patient’s maternal grandfather, father, maternal uncle, and younger brother were within the normal range. The Lyso-GL-3 level of the patient was slightly elevated above the normal range; the Lyso-GL-3 test was not performed for the other family members. Whole-exome sequencing revealed that the patient carried a hemizygous variant of the *GLA* gene (c.735G>C). His mother and maternal grandmother were heterozygous carriers of the same variant. (**Figure 2**) According to the American College of Medical Genetics and Genomics guidelines, this variant is classified as uncertain significance (VUS). However, another pedigrees with renal phenotypes harboring this variant have been identified by laboratories specializing in Fabry disease diagnostics(not yet published). Consequently, the evidence has been upgraded to “likely pathogenic”(PS3_moderate+PM1+PP3+PM2_supporting).

## 7 Treatment and follow-up

**Figure 3:**
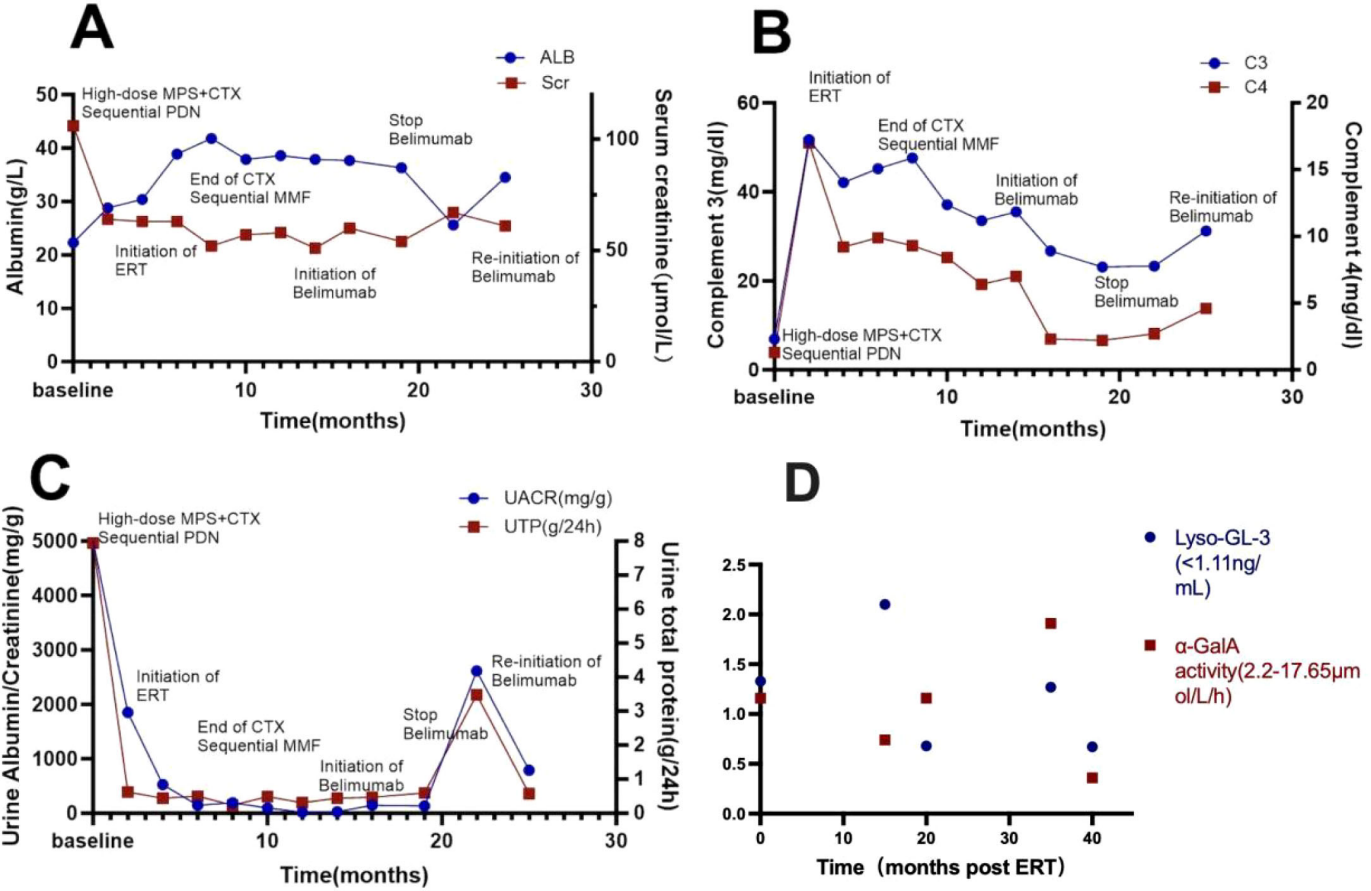
Dynamic changes in patient treatment and biochemical indicators including albumin, serum creatinine **(A)**, complement C3, complement C4 **(B)**, urine albumin/creatinine ratio and 24-hour urinary total protein **(C)**.Irregular monitoring values of biomarkers post-ERT treatment **(D)**.

The patient with SLE and was initially treated with methylprednisolone pulse therapy, followed by oral prednisone and low-dose intravenous cyclophosphamide, which was then transitioned to oral mycophenolate mofetil.Losartan was used to manage proteinuria. After confirming FD genetically, enzyme replacement therapy(ERT) with agalsidase-α was initiated in addition to the existing treatment regimen. Two months later, proteinuria resolved, and prednisone was reduced to 5 mg daily. Due to low complement levels, belimumab was introduced but discontinued after no improvement post 8 infusions. In October 4, 2024, the patient had recurrent fever and nephrotic-range proteinuria, leading to the reintroduction of belimumab. Follow-up showed proteinuria at 500 mg/day, serum albumin at 39 g/L, and persistently low complement levels. The patient experienced the infusion-related adverse reaction during the observation period after completing the full infusion of agalsidase. Analysis indicated the reaction was associated with the excessive infusion speed. Symptoms were promptly alleviated following immediate treatment. Therefore, after two weeks, agalsidase therapy was resumed with strictly controlled infusion rates.The seemingly ‘random’ (fluctuating or irregular) follow-up results of α-Gal A activity and lyso-GL3 levels may reflect the disease’s complex pathophysiology and multifactorial interference (**Figure 3**).

## 8 Literature review

**Figure 4:**
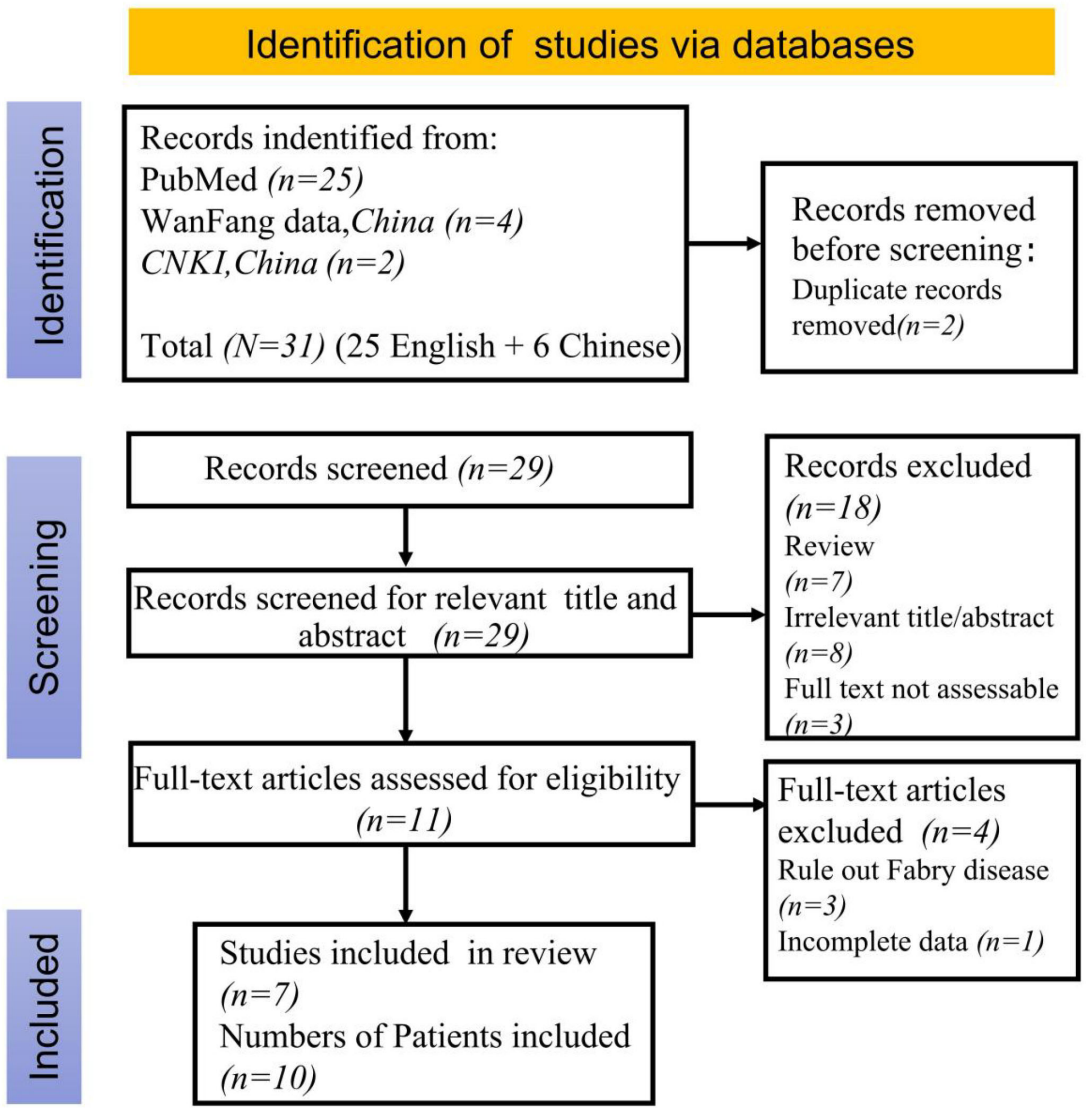
Flow chart of literature selection according to PRISMA guidelines.

A literature search using the keywords “systemic lupus erythematosus”, “Fabry disease” and “GLA deficiency” was conducted on PubMed,Wanfang Database and China National Knowledge Infrastructure (CNKI) up to February 2025, excluding review articles. This search yielded 1 Chinese and 6 English articles. (**Figure 4**) Along with a current case study, these articles helped summarize the clinical and genetic profiles of 11 SLE patients with FD (9–18) (**Table 1**). Among them, 2 were males and 9 females, with a median FD diagnosis age of 36 years (range: 12–61 years); 3 were diagnosed before 18. Key symptoms included lupus nephritis (9 patients), rash, alopecia, oral ulcers (8), arthritis (7), cardiac issues (4), fever (4), Raynaud’s phenomenon (1), and corneal lesion (1). All tested positive for ANA or lupus cells. Renal biopsies were done on 9 patients, and myocardial biopsies on 2.

**Table 1:** Clinical features in patients with fabry disease overlapping with systemic lupus erythematosus.

Study	Published year	Reign	Sex/age(y) at diagnosis of FD	Main symptoms	SLE-related lab tests	Biopsy(renal/heart)	Treatment	Genetic testing	GLA activity (nmol/h/mL)	Lyso-Gb3c (nmol/L)
Loeb etal (9)	1968	Arab	M/16	fever, arthralgia, facial rash, hematuria, decreased urea clearance	LE cell(+)	Fabry nephropathy	Not reported	NA	partial deficiency	NA
Rosenmann etal (10)	1983	Arab	F/15	fever, joint pains, skin rash,proteinuria	ANA,LE cell(+)	active focal lupus glomerulonephritis and visceral epithelial cell cytoplasm containing osmiophilic multilamellar lipoid bodies	GCs	NA	partial deficiency	NA
Majima etal (11)	1992	Japan	F/36	nephrotic syndrome	ANA1:640, anti-dsDNA(+), hypocomplementaemia	diffuse membranous glomerulonephritis and vacuolization of epithelial cells;subepithelial and subendothelial dense deposits and visceral epithelial cell cytoplasm containing osmiophilic multilamellar lipoid bodies	Not reported	NA	partial deficiency	NA
Rahman etal (12)	1998	Egypt	F/49	ephitelial keratopaty,corneal teleangectasia,arthritis,chest pain palpitation,cardiac congestive failure	NA	Fabry nephropathy	GCs;HCQ	NA	NA	NA
Waggoner etal (13)	2010	USA	F/36	Raynaud phenomenon, arthralgia, photosensitive rash, proteinuria, dry eyes and dry mouth, alopecia,depression	ANA1:160, anti-U1RNP(+)	foamy vacuolation with osmiophilic lamellated electron-dense concentric myelin-like bodies in the glomerular podocytes, tubular epithelium, and interstitial cells	HCQ;nortriptyline; pregabalin;duloxetine	GLA : NM_000169.3:exon7:c.G1025A:p.Arg342Gln	low	NA
Ma Ya etal (14)	2012	China	F/58	limbpain,rash,breathlessness,LVH, arthralgia, rampant tooth decay,proteinuria	ANA 1:1280, anti-SSA,anti- SSB(+)	lupus nephritis IIIA;diffuse foamy degeneration of podocytes in glomerulus and medullary body	GCs;CTX;aspirin; anticoagulants	GLA : NM_000169.3:exon2:c.C334T:p.Arg112Cys	normal	NA
Nandagudi etal (15)	2013	Caucasian	F/38	chest pain with shortness of breath;arthralgia;loose stools; photosensitivity;malar rash; occasional mouth ulcers, increased sweating,LVH	ANA1:640, anti-dsDNA(+),aPL(+), hypocomplementaemia	sarcoplasmic vacuolation and myofibrillary loss of myocytes;electron-dense glycosphingolipid in the form of myelin figures in the myocyte sarcoplasm.	HCQ;GCs;azathioprine; Fabrazyme;atorvastatin;anticoagulants;diltiazem;ERT	GLA : NM_000169.3:exon7:c.C1028T:p.Pro343Leu	normal	NA
Chatre etal (16)	2016	France	F/61	alopecia, skin rash, joint pain, LVH, persistent weakness,chest pain with breathing difficulties	ANA1:100	cytoplasmic vacuolization of cardiomyocytes and myelin figure inclusions,glycogen accumulation in cardiomyocytes	GCs;HCQ	GLA : NM_000169.3:exon2:c.T337C:p.Phe113Leu	low	NA
Conigliaro etal (17)	2020	Italy	F/45	acute renal failure	ANA1:640, anti-dsDNA(+), hypocomplementaemia	lupus nephritis IV A/C with chronic tubular interstitial damage	GCs;CTX;MMF;HCQ, IVIG;kidney transplantation	GLA : NM_000169.3:exon3:c.A376G:p.Ser126Gly	NA	normal
Celia etal (18)	2021	Italy	F/23	fever, oral ulcers, effluvium, asthenia,arthromyalgia, proteinuria	ANA 1:160, anti-dsDNA(+), hypocomplementaemia	extensive vacuolization with foamy appearance of podocytes;cytoplasmic lamellar bodies	GCs;MMF;HCQ; ramipril,ERT	GLA : NM_000169.3:exon2:c.G335A:p.Arg112His	NA	NA
This study	2025	China	M/12	fever,hematuria, proteinuria	ANA 1:1000; anti-dsDNA, anti-SSA, anti-SSB, anti-Ro52(+); hypocomplementaemia	active lupus glomerulonephritis IV-A+V;cytoplasmic swelling and vacuolar degeneration in capillary endothelial cells and podocytes; a few myeloid bodies in podocytes	GCs;CTX;MMF; losartan, belizumab,ERT	GLA : NM_000169.3:exon5:c.G735C:p.Trp245Cys	low	low

LE, Lupus Erythematosus; ANA, Anti-Nuclear Antibody; anti-dsDNA, Anti-Double Stranded DNA Antibody; anti-SSA, Anti-Sjögren’s Syndrome A Antibody; anti-SSB, Anti-Sjögren’s Syndrome B Antibody; anti-Ro52, Anti-Ro52 Antibody; anti- U1RNP, Anti-U1 Ribonucleoprotein Antibody; aPLs, Anti-Phospholipid Antibodies; LVH, Left Ventricular Hypertrophy; GCs, Glucocorticoids; HCQ, Hydroxychloroquine; CTX:cyclophosphamide; ERT, Enzyme Replacement Therapy; MMF, Mycophenolate Mofetil; IVIG, Intravenous Immunoglobulin; NA, Not Applicable.

The *GLA* gene mutations were mostly hemizygous in males and heterozygous in females and involved 7 mutation sites, namely c.G1025A:p.Arg342Gln, c.C334T:p.Arg112Cys, c.C1028T:p.Pro343Leu, c.T337C:p.Phe113Leu, c.A376G:p.Ser126Gly, c.G335A:p.Arg112His, and c.G735C:p.Trp245Cys. The treatment mainly included glucocorticoids, with 2 patients treated with cyclophosphamide, 2 patients with mycophenolate mofetil, 3 patients with ERT, and 1 patient with belimumab therapy.

## 9 Discussion

FD is a rare and complex condition primarily affecting the kidneys and often coexists with other hereditary or acquired kidney diseases such as IgA nephropathy, membranous nephropathy, IgM nephropathy, Alport syndrome (19–22). Although each case is rare, they highlight the diagnostic challenges FD poses, suggesting it should be considered in kidney disease evaluations. The present case involves an adolescent male with multisystem issues, including kidney, lung, and serous cavity effusions. Positive autoantibodies and low complement levels indicated SLE, with initial kidney pathology confirming lupus nephritis. Electron microscopy showed myeloid bodies, ruling out drug-induced changes mimicking FD. FD was diagnosed through enzymatic activity and genetic testing, which was conducted on the admission day, allowing for timely treatment. Both FD and SLE can cause multiorgan damage with overlapping symptoms. FD is a monogenic disorder, while SLE is typically polygenic, though some cases are due to single-gene mutations. These diseases differ in their pathogenic mechanisms and renal manifestations.So far, only a few coexisting cases have been reported globally. Emerging evidence suggests a potential link between FD and SLE. Rosenmann et al (10) first reported a case of coexisting FD and SLE, proposing that accumulated glycosphingolipids may trigger autoimmunity. Subsequent studies found a high prevalence of autoantibodies in FD patients (23), though no FD cases were detected in pediatric SLE screening (24). Mechanistically, Gb3 accumulation may stimulate lymphocytes, promoting autoantibody production (12). Because of the primary pathological changes caused by FD, some patients may exhibit increased susceptibility to SLE, with the degree of susceptibility potentially influenced by various genetic and/or nongenetic factors. Further research is required to elucidate the link between the two diseases.

Sphingosine-1-phosphate (S1P), a central bioactive lipid in sphingolipid metabolism and a critical regulator of lymphocyte trafficking, is closely associated with autoimmune diseases, including SLE (25). It plays a pivotal role in regulating various pathophysiological processes, including cell proliferation, survival, migration, synthesis of inflammatory mediators, and tissue remodeling (26).As shown previously, SLE patients and lupus mouse models exhibit elevated S1P levels (27, 28), which exacerbate disease activity and organ damage. These studies support the hypothesis that Sphingomyelin signaling pathway may be involved in the pathogenesis of both FD and SLE.

SLE management follows treat-to-target and personalized pharmacotherapy, using antimalarials, glucocorticoids, immunosuppressants, and targeted biologics. For refractory SLE in children, stem cell transplantation and CAR T-cell therapy are options (29). FD treatment typically involves intravenous ERT and oral chaperone therapy, with new therapies like plant-based ERT (pegunigalsidase alfa and moss-aGal), substrate reduction, mRNA and gene therapy under investigation. Most patients with both SLE and FD receive glucocorticoids and immunosuppressants, though early-diagnosed patients often miss ERT due to medication shortages. In the current case, the patient was treated with glucocorticoids, cyclophosphamide, mycophenolate mofetil, losartan, agalsidase-α, and belimumab. Several aspects of this treatment course were perplexing and deserve further discussion.

### 9.1 HCQ treatment was discontinued

HCQ is widely used to treat SLE due to its immunomodulatory and anti-inflammatory properties, effectively reducing disease activity and improving long-term prognosis (30). It is recommended for all cSLE types (31). However, HCQ can cause renal changes similar to FD, known as drug-induced phospholipidosis (DIPL), often misdiagnosed as FD (32–34). While HCQ use is generally discouraged during ERT for FD, its use in patients with both SLE and DIPL exclusion is debated. A recent study (35) conducted cellular and animal experiments to investigate whether HCQ affects the efficacy of ERT. The results showed that HCQ at low concentrations had a minimal direct effect on the enzymatic activity of α-Gal A and did not affect the catalytic activity of agalsidase-β toward Gb3/Lyso-Gb3 in the liver, kidneys, and heart of FD model mice; however, cultured cells treated with HCQ exhibited reduced enzymatic activity of α-Gal A. Among the 11 reported patients with concomitant SLE and FD, HCQ treatment was administered in 6 patients; however, it remains unclear whether HCQ treatment was discontinued due to a lack of data. In this case, HCQ was halted after a renal biopsy showed myeloid bodies, and since it was used for just one day, drug-induced changes were ruled out. Research typically uses mice, not humans, so the effects of HCQ in SLE and FD patients are still uncertain and need further investigations.

### 9.2 Effects of ERT on proteinuria

Since 2001, ERT has been used to treat FD by addressing α-Gal A deficiency and reducing cellular substrate buildup. It has become more common in clinical settings, showing positive effects on cardiac and renal functions and neuropathic pain relief (36–38). However, its impact on proteinuria is unclear, as some studies suggest ERT alone may not reduce it (39–41).

Podocytes are highly specialized and differentiated cells that cover the outer surface of glomerular capillaries and play a crucial role in maintaining the structure and function of the glomerular filtration barrier. Dysfunction or loss of podocytes is closely associated with proteinuria and represents a key factor in the development and progression of renal disease in patients with FD. Podocytes with a deficiency in α-Gal A activity exhibit phenotypic characteristics related to hypertrophy, fibrosis, increased vascular reactivity, epithelial-mesenchymal transition, defects in autophagy and the ubiquitin-proteasome system, and apoptosis (42). Glomerular podocytes are relatively resistant to ERT and have poor proliferative capacity, with minimal ability to compensate for cell loss (43). An interesting study (44) found that treatment with agalsidase-β at 1.0 mg/kg/every other week (eow) partially cleared the accumulated GL3 in podocytes. However, after switching to agalsidase-α at 0.2 mg/kg/eow for 3 years, GL3 reaccumulated in podocytes. Subsequently, after switching back to agalsidase-β at 1.0 mg/kg/eow for 2 years, the GL3 levels in podocytes again decreased. This finding indicates that the type of ERT also affects the clearance of accumulated GL3 in podocytes. Additionally, previous studies revealed that supplementation of α-Gal A activity and clearance of accumulated Gb3 are insufficient to rescue the dysregulation of profibrotic signaling pathways and autophagy in *in vitro* cultured models of podocytes from FD patients (40). Moreover, the efficacy of ERT may vary depending on the patient’s sex and genotype, timing of treatment initiation, drug dosage, and disease progression stage. Personalized treatment regimens and combination with adjunctive therapies may be the key approaches to improve therapeutic outcomes.

### 9.3 Persistently low complement levels throughout the treatment course

Belimumab, a monoclonal antibody targeting the B-lymphocyte stimulator, is vital in SLE pathogenesis and can reduce disease activity markers and complement levels (45–47). Current guidelines primarily recommend belimumab for SLE patients with autoantibody-positive status and active clinical manifestations. In this refractory case, we opted for belimumab therapy with dual objectives: to facilitate glucocorticoid tapering for sustained remission, while simultaneously monitoring for potential improvement in hypocomplementemia. Despite aggressive pharmacotherapy, persistent hypocomplementemia was observed.Let us now return to the discussion of Fabry disease.FD is characterized by the progressive accumulation of Gb3 and related glycosphingolipids in vascular endothelial cells. This pathological accumulation of Gb3 triggers a series of processes involving immune dysregulation and activation of the complement system. Several research studies have highlighted the effect of immune dysregulation on FD, with particular emphasis on the complement system. A South Korean study (48) used two-dimensional electrophoresis, matrix-assisted laser desorption/ionization-time of flight tandem mass spectrometry, and tandem mass spectrometry to analyze and compare the plasma proteomics of eight patients with classic FD before and after ERT. The study found that the levels of inactivated complement C3b (iC3b) and C4B were significantly elevated in the plasma of FD patients before ERT. Following short-term ERT (4–12 months), the levels of C1QC, C3, and C4 were significantly reduced. After long-term ERT (46–96 months), the levels of iC3b gradually decreased, and this reduction was comparable to the decrease in Gb3 levels. Additionally, substantial deposition of C3 was observed in renal tissues of patients with FD prior to treatment. In the present case, C3 and C4 levels also exhibited a downward trend following ERT treatment, which may have influenced the efficacy of belimumab therapy. According to a recent study, complement activation in patients with FD is robust and independent of ERT. This is particularly evident in males with nonsense mutations and anti-drug antibodies. Despite ERT, cellular activation and inflammatory responses persist in FD patients. This ongoing chronic inflammation can promote organ damage (49). The extent to which ERT influences and modulates complement system activation remains unclear. The complement system could play a critical role as an inducer of FD-related nephropathy and could potentially serve as a useful intervention target for preventing progressive organ damage.

## 10 Conclusion

This case demonstrates the diagnostic and therapeutic challenges in managing concomitant Fabry disease and systemic lupus erythematosus. While belimumab showed efficacy for SLE manifestations and enzyme replacement therapy helped preserve renal function, several limitations must be acknowledged: the 27-month follow-up precludes long-term prognostic assessment, unavailable drug resistance data, and the exceptional rarity of such cases restricting comparative analyses. Emerging therapies targeting the complement system may have the potential to reduce inflammation and mitigate tissue damage, thus offering promising prospects for future applications.

## Data Availability

The original contributions presented in the study are included in the article/supplementary material. Further inquiries can be directed to the corresponding author.
